# Development of an LC-MS method for the determination of simvastatin and its hydroxy acid form in muscle tissue and method application

**DOI:** 10.1371/journal.pone.0322808

**Published:** 2025-05-05

**Authors:** Ewa Paszkowska, Karolina Pietrowska, Steen Larsen, Emilia Fornal, Michał Ciborowski

**Affiliations:** 1 Medical University of Lublin, Department of Bioanalytics, Lublin, Poland; 2 Medical University of Bialystok, Metabolomics and Proteomics Laboratory, Clinical Research Centre, Bialystok, Poland; 3 Department of Biomedical Sciences, Faculty of Health and Medical Sciences, University of Copenhagen, Xlab, Center for Healthy Aging, Copenhagen, Denmark; University of Lucknow, INDIA

## Abstract

**Purpose:**

Statins are the most commonly used drugs worldwide. Besides a significant decrease in cardiovascular diseases (CVDs) risk, the use of statins is also connected with a broad beneficial pleiotropic effect. At the same time, it is burdened with different side effects. The most common ones are muscle issues (from mild myalgia to rhabdomyolysis). The mechanisms of many of them are still unclear. Therefore, an analytical method for the determination of simvastatin (SIM) and its main metabolite (the hydroxy acid form – SIMA) in muscle tissue was developed.

**Methods:**

Muscle samples were homogenized with ammonium acetate buffer using the bead mill homogenizer, and then statins were extracted with a mixture of methanol and ethanol. Prepared samples were analyzed with liquid chromatography (using a reverse-phase column with a gradient elution) combined with the mass spectrometer which was operated in a multiple reaction monitoring mode.

**Results:**

The assay was linear over a 0.1–5 ng/mL range for both statin forms. Inter- and intra-day precision and accuracy were characterized. The method was considered precise (with the following relative standard deviation values: 6.0–6.9% for SIM, and 8.1–12.9% for SIMA) and accurate (with the following mean accuracies: 91.4–100.1% for SIM, and 102.2–115.4% for SIMA). The extraction efficiency was evaluated by recovery determination (76% for SIM, and 99% for SIMA). Moreover, the matrix effect was calculated with the following results: 87% for SIM, and 139% for SIMA. The proposed method was applied for SIM and SIMA determination in skeletal muscle tissues obtained from statin-treated patients.

**Conclusion:**

The obtained results proved that the method may be a useful tool for explaining muscle effects related to statin therapy.

## 1. Introduction

Statins belong to medicines that are most commonly prescribed worldwide [1–4]. They are successfully used in therapy for primary and secondary prevention of cardiovascular incidents [2,5]. A high level of low-density lipoprotein (LDL) cholesterol is one of the main risk factors for cardiovascular diseases (CVDs) [6]. In general, statins, through the inhibition of 3-hydroxy-3-methylglutaryl coenzyme A (HMG-CoA) reductase in the mevalonate pathway, decrease the LDL level and help to control it [3,7].

Simvastatin (SIM) has a relatively low plasma concentration because of the efficient first-pass hepatic extraction [8,9]. The efficiency of this phenomenon is a result of SIM lipophilic properties. This statin type crosses the hepatic cell membrane on the passive diffusion way [7,8]. This method of introduction into the cell also allows getting inside the extrahepatic tissue because it is a non-selective method for any specific cell type [7,10]. SIM is an inactive lactone (the prodrug form) converted into a few active metabolites in the liver [11,12]. The hydroxy acid (simvastatin hydroxy acid – SIMA) is the most noteworthy form. It is also a more polar and pharmacologically active form, which indeed impacts the mevalonate pathway [11–14].

**Fig 1:**
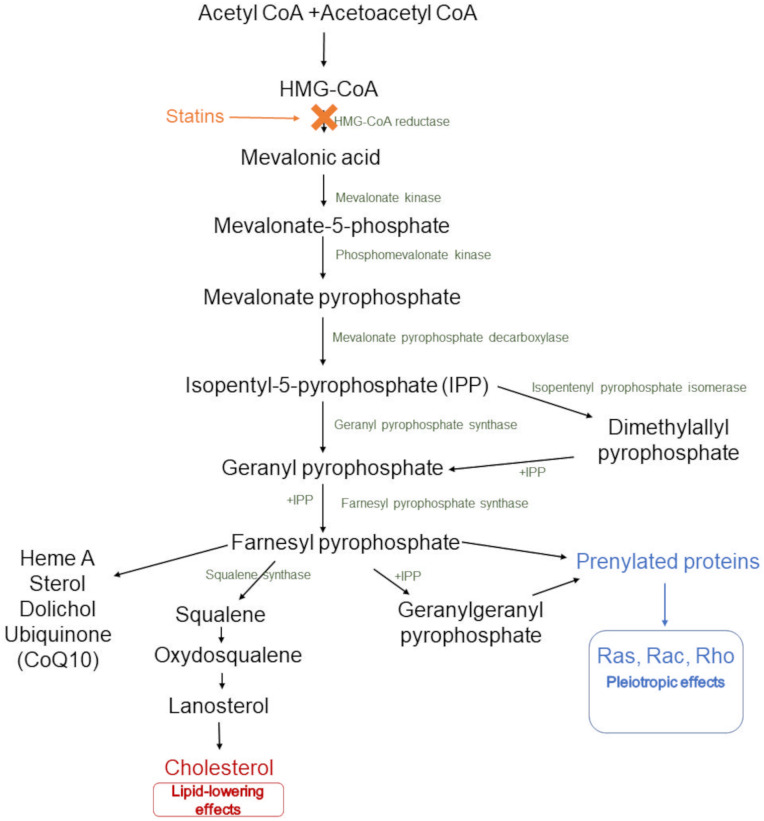
The mevalonate pathway (cholesterol biosynthesis): the place of action of statins, and their main effects.

The mevalonate pathway (Fig 1) is the main target of statin interaction during LDL-lowering therapy. As a result of the mevalonate synthesis inhibition, the level of many important metabolites included in this pathway (e.g., farnesyl pyrophosphate, geranylgeranyl pyrophosphate, or ubiquinone) decreases [7]. This may be one of the reasons for the pleiotropic effects of statin therapy. Many articles reporting beneficial effects of statin therapy other than CVDs prevention (*via* decreasing the LDL level) were summarised in a few interesting reviews [7,15–18]. As shown there, the use of statin may exert, inter alia, anti-oxidant, anti-inflammatory, anti-fibrotic, and neuroprotective effects, improve cardiovascular and renal functions, as well as enhance bone formation [17]. The pleiotropic effect of statin therapy was observed in endothelial cells, vascular smooth muscles, myocardium, platelets, and brain cells [7,18]. Moreover, Oesterle et al. [16] pointed out a few mechanisms connected with this additional beneficial effect of statin therapy. Influence on the isoprenylated proteins, Rho/Rho kinase, Rac protein, or peroxisome proliferator-activated receptor (PPAR) was described as a potential mechanism. However, the term pleiotropic includes not only beneficial effects. It is also well-known that many adverse effects are connected with statin therapy [1,2,19,20]. The most common side effects refer to muscles (a broad spectrum of effects, from myalgia and myopathy to rhabdomyolysis) [1,2,5,19–22], including an increased incidence of new-onset diabetes and serious liver injuries [19,21]. Additionally, ambiguous results are presented for the correlation of statin use with decreased renal function, tendon rupture, hemorrhagic stroke, interstitial lung disease, lower testosterone, depression, memory loss, cataracts, and sleep disturbances [1,2,19,21].

The main aim of the presented research was to develop a sensitive analytical method that allows the determination of SIM and SIMA concentrations in muscle tissue. Because of its high selectivity and sensitivity, a combination of liquid chromatography with a triple quadrupole mass spectrometer (LC-MS) was chosen as an analytical tool. The proposed method was applied to determine SIM and SIMA in the muscle samples obtained from the statin-treated patients (with hypercholesterolemic patients without statin treatment as the control group).

The selective and sensitive LC-MS coupling is suitable for determining the statin levels in different biological material types. Analytical methods allowing the quantification of statin in plasma samples are broadly described [9,11,13,14,23,24]. However, only two publications present an LC-MS application for the quantitative determination of statins in solid tissue. Christiansen et al. [25] measured atorvastatin, and its two metabolites, in different sample types, including liver, heart, and skeletal muscle. Mucha et al. [26] used the LC-MS systems to determine simvastatin in muscle tissue, but without describing the details of the method. The lack of these details makes it impossible to compare the presented here methods efficiency or repeat these measurements in other laboratories. Consequently, this is the first study presenting the development of the LC-MS method for determining simvastatin and its hydroxy acid form in muscle tissue. Determination of the metabolites mentioned above in the tissue of SIM-treated individuals may help to understand the mechanisms of beneficial and adverse statin therapy effects.

## 2. Materials and methods

### 2.1. Chemicals and reagents

Purified water was obtained using the Milli-Q Integral 3 system (Millipore SAS, Molsheim, France). SIM (the analytical standard 99%), lovastatin (LOV; PhEu reference standard), which was used as one of the internal standards (IS), ethanol (EtOH; LC grade), acetic acid (PhEu reagent grade), ammonium acetate and formic acid (LiChropur, an eluent additive for LC-MS), and tert-butyl methyl ether (MTBE; LiChrosolv, for liquid chromatography) were purchased from Sigma-Aldrich Chemie GmbH (Steinheim, Germany). Lovastatin hydroxy acid sodium salt (LOVA, analytical standard 97%), which was an additional IS, and SIMA (the analytical standard 99%) were purchased from Biosynth Ltd (the United Kingdom). Methanol (MeOH), acetonitrile (ACN), and isopropanol, all the Optima LC-MS grade, phosphate-buffered saline (PBS) tablets, and ethyl acetate (EtAc) were all purchased from Thermo Fisher Scientific (San Jose, California, the USA).

### 2.2. Study group

The study was approved by the Ethics Committee of the Medical University of Bialystok (APK.002.331.2022). The study was conducted based on the archival biological material (samples were collected between January 2015 and December 2016). The authors had no access to information that could identify individual participants. Data was generated between the 7^th^ of July 2023 and the 25^th^ of October 2023 based on the collected samples. After that step till the 6^th^ of March 2024, these data were analyzed. Samples were obtained from hypercholesterolemic patients either at the diagnosis moment, before starting the statin treatment (control group), or already statin-treated patients with stabilized state – whose blood parameters and blood pressure reached therapeutic goals and presented correct values (study group). The daily dose of simvastatin was 40 mg. Skeletal muscle samples (*vastus lateralis*) were obtained from 86 patients (the detailed group characteristic is in Table 1) with the Bergstrom needle biopsy technique modified for suction under local anesthesia (Lidocaine 5%). After the procedure prepared samples were placed at -80°C. From the same patients, blood samples were collected. Based on these samples SIM and SIMA in plasma were determined. The method for SIM and SIMA determination in plasma samples is described in the supporting information (S1 Appendix).

**Table 1:** The characteristics of patient groups were used to prove the method’s applicability.

Parameter	Statin treatment	Control	*p*-value
N	73	13	–
Gender (F/M)	24/49	5/8	0.90
Age [years]^a^	62 ± 5.5	59 ± 7.3	0.26
BMI^a^	28 ± 3.3	28 ± 2.5	0.51
Cholesterol [mM]^a^	4.19 ± 0.60	6.02 ± 1.05	<0.01
LDL [mM]^a^	2.47 ± 0.59	4.32 ± 0.96	<0.01
Systolic blood pressure [mm Hg]	136 ± 14.4	128 ± 16.2	0.07
Diastolic blood pressure [mm Hg]	84 ± 9.4	83 ± 9.4	0.55
SIM in plasma [ng/mL]^a^	1.52 ± 1.05	<LOQ	–
SIMA in plasma [ng/mL]^a^	3.43 ± 2.68	<LOQ	–

^a^Mean value ± SD.

### 2.3. LC-MS instrumentation and conditions

The LC-MS system was composed of a 1290 Infinity II UHPLC system, combined with a 6495 triple quadrupole mass spectrometer (both from Agilent Technologies, Santa Clara, California, the USA) equipped with the electrospray ionization (ESI AJS) ion source and iFunnel technology.

#### 2.3.1. Optimized chromatographic conditions.

Chromatographic separation was performed on an analytical column Zorbax RRHD Eclipse Plus C18 (2.1 × 50 mm, 1.8 µm), connected to a guard column (Zobrax RRHD Eclipse Plus C18, 2.1 × 5 mm, 1.8 µm), thermostated at 45°C. Column type was selected based on the literature [11,13,14,25,27].

Isocratic and gradient elution were tested. Different gradient elutions with various mobile phase compositions were used to select the chromatographic conditions that provide the best peak separation. Water with 0.1% formic acid addition or ammonium acetate buffers (2 mM, 10 mM, 50 mM, and 100 mM) was used as phase A. While pure ACN, ACN with 0.1% formic acid addition, and ACN/isopropanol (6:4, v/v) mixture were used as phase B. Mobile phase compositions and elution types were selected for testing based on the literature [11,13,14,27].

#### 2.3.2. Final LC-MS conditions.

The gradient with the ammonium acetate buffer (50 mM in water, pH 5.0 adjusted with acetic acid) as phase A, and pure ACN as phase B, was used to separate statins and matrix components as follows: the initial 5% B was increased to 60% B in 2 min, and then further to 85% B in 3 min, to reach the final condition of 95% B at 5.2 min, and was then retained for 3.8 min (total run time: 9 min). After each run, the system was conditioned for 5 min with the initial mobile phase composition. The flow rate was 0.25 mL/min. The autosampler was maintained at 8°C, and the injection volume was 5 µL. The mass spectrometer was operated in the multiple reaction monitoring mode (MRM) with switching polarization. SIM and LOV were observed in the positive ion mode, while SIMA and LOVA were in the negative ion mode. The detailed information about *m/z* transitions precursor/product ions is presented in Table 2. The ion source conditions were as follows: the gas temperature was set at 290°C with a 15 L/min gas flow rate, and the sheath gas temperature and flow rate at 350°C and 11 L/min, respectively. The nebulizer worked at 40 psi, the nozzle voltage was 0 V, and the capillary voltages were 3000 V for the positive ion mode and 5500 V for the negative ion mode.

**Table 2:** The observed MRM reactions.

Compound	Precursor ion	Product ion	Collision energy [V]	Retention time [min]
SIM	419.1*	199.2*	30	4.80
	419.1	225.2	32	
	419.1	303.3	12	
SIMA	435.0*	319.0*	20	3.05
	435.0	115.0	14	
LOV	405.2*	285.1*	10	4.35
	405.2	303.2	10	
	405.2	199.2	20	
LOVA	420.8*	319.0*	18	2.87
	420.8	101.0	40	

*) Precursor/product ion transition used as quantifier.

### 2.4. Preparation of standards solutions

Stock solutions of SIM, SIMA, LOV, and LOVA (2 mg/mL) were prepared by dissolving the accurately weighed standards in methanol. Working solutions (WS) were prepared separately for SIM and SIMA (one series of solutions) and for LOV and LOVA (which worked as an IS solution). The stock standard solutions of SIM and SIMA were diluted with a MeOH/EtOH (1:1, v/v) mixture to achieve

the standard WSs at the following concentrations: 1, 5, 10, 100, and 500 ng/mL. LOV and LOVA stock solutions were also diluted with the same mixture of MeOH/EtOH to achieve a concentration of 500 ng/mL. Additionally, the mixture of all four standards diluted in PBS to 1 µg/mL was prepared for method optimization purposes.

### 2.5. The procedure of muscle tissue sample preparation

The experiment was divided into two parts: optimization and validation. Pig skeletal muscle tissue was used as a sample matrix for both of them. In the first part, solutions for homogenization and extraction were selected, and the solvent-to-tissue weight proportion was optimized. Four solvents for homogenization were tested, i.e., MTBE, EtAc, ACN, and the MeOH/EtOH (1:1) mixture. These solvents for the test were selected based on the optimization procedures of SIM and SIMA extraction from plasma samples described in the literature [13,27]. To reduce the interconversion of SIM and SIMA forms (which is pH-dependent) in samples before analysis the ammonium acetate buffer (0.5 M) was additionally tested as a homogenization solution. The same solutions (with the exclusion of the ammonium acetate buffer) were also used as extraction agents. Another optimized parameter was the volume of the homogenization and extraction solutions used per tissue weight. To obtain the most efficient homogenization and extraction, three volumes were tested: 5, 10, and 25 µL of the solvent or solvents mixtures per 1 mg of tissue.

#### 2.5.1. Preparation procedure of samples used for the method optimization.

The tissue samples used for method optimization were prepared by incubating tissue portions for 2 hours at 4°C in PBS containing all four standards (at 1 µg/mL). After incubation, the samples were placed overnight in a freezer (–80°C). On the day of analysis tissue samples were weighted and placed in Eppendorf tubes with four stainless steel beads (3 × 3 mm and 1 × 5 mm) and the homogenization solution was added. Then, the tissue samples were homogenized using a bead mill homogenizer (Tissue Lyser LT; Qiagen Hilden, Germany) for 5 min (50 Hz). After homogenization, the extraction solvent was added in an equal volume to the homogenization solvent. The samples were then vortex-mixed for 5 min and incubated on ice for 30 min. After the extraction step, beads were removed, and the samples were centrifuged at 21,000 × g for 20 min at 4°C. 300 µL of the supernatant was transferred into glass vials and evaporated to complete dryness using a vacuum concentrator (SpeedVac, Thermo Fisher Scientific, San Jose, California, the USA). Then, the samples were reconstituted in 300 µL of the H_2_O/ACN (7:3, v/v) mixture, filtered through a 0.22 µm nylon filter, and analyzed using an LC-MS system.

#### 2.5.2. Preparation procedure of real samples and samples used for validation.

On the day of analysis, samples were weighed and placed in an Eppendorf tube. The range of the weight of samples collected from patients was 10.23–22.68 mg (with a median equal to 17.79 mg). In the validation part, blank tissue (without any pre-treatment) was used. Then, four stainless steel beads (3 × 3 mm and 1 × 5 mm), 0.5 M ammonium acetate buffer (in the role of homogenization solvent) in proportion 5 µL per 1 mg of sample, and IS (0.05 ng per 1 mg) were added. Additionally, in the samples used for validation, the WSs were spiked. These mixtures were homogenized for 5 min (50 Hz) in the bead mill homogenizer. After that step, for statin extraction, the MeOH/EtOH mixture in the amount of 5 µL per 1 mg of sample was added, then samples were vortex-mixed for 5 min, and incubated on the ice for 30 min. In the next step, steel beads were removed, and samples were centrifuged for 20 min at 4°C at 21 000 × g. After centrifugation obtained supernatants were filtered through a 0.22 µm nylon filter into glass vials and analyzed using the developed LC-MS method.

### 2.6. Calibration curve and quality control samples

To prepare the samples for the calibration curve, statin-free tissue samples were spiked with the WS containing SIM and SIMA at the following concentrations: 0.1, 0.2, 0.5, 1, 2, and 5 ng/mL. A quality control (QC) sample containing SIM and SIMA at a concentration of 5 ng/mL was prepared separately from the samples used to obtain the calibration curve. Both series (the calibration and QC samples) were also spiked with the IS at a concentration of 5 ng/mL. All these samples were processed as described above.

### 2.7. Method validation

The method was validated according to the ICH guideline M10 on bioanalytical method validation [28], and the FDA guidelines [29]. The assay was validated for selectivity, linearity, precision, accuracy, extraction recovery, and the matrix effect.

#### 2.7.1. Selectivity and specificity.

The selectivity of the method was evaluated based on analyzing blank muscle tissue and tissue spiked with both forms of the statin and IS. Samples were processed with the previously described procedure (the blank muscle sample was processed without the addition of the WS or the IS), and analyzed with the prepared LC-MS method. Selectivity was checked by visual evaluation of the chromatogram, comparing the blank peak responses with the peak response for the lower limit of quantification (LOQ). The LOQ was defined following the FDA recommendation [29] as the lowest concentration of analyte that could be measured with precision below 20%, and accuracy in the range of 80–120% calculated based on the constructed regression model. The sample with standards spiking was used for evaluation of the specificity of the method.

#### 2.7.2. Linearity.

The linearity of the method was evaluated using the blank muscle tissue spiked with SIM and SIMA in the concentration range of 0.1–5 ng/mL, and both the ISs (LOV and LOVA) at 5 ng/mL in each of the calibration samples. These samples were prepared in triplicate and each calibration series was analyzed on three different days. The ratios of the area of the response of the analyte to the IS were plotted against the concentration of each calibration standard, and the obtained results were fitted into a linear regression model.

#### 2.7.3. Precision and accuracy.

The inter- and intra-day precision and accuracy were evaluated by analyzing the tissue samples spiked with SIM and SIMA at three concentration levels: 0.2 ng/mL, 1 ng/mL, and 5 ng/mL. To obtain the parameters describing an inter-day assay, the samples were analyzed four times during an analytical run. Similarly, the intra-day assay was performed by analyzing these samples over four days. The data met the acceptance criteria when the standard deviation of the calculated concentration was ≤ 15% of the nominal concentration (accuracy), and the relative standard deviation (RSD) was also ≤15% (precision) [29].

#### 2.7.4. Carry-over evaluation.

Samples were tested to determine potential carry-over between injected samples. After analysis of the calibration samples (with increased concentration), the blank samples series was injected. The residue of analytes was evaluated by comparing signals obtained from blank samples versus LOQ level. Carry-over acceptance criteria is less than 20% for SIM and SIMA, and less than 5% for IS (LOV and LOVA) [28].

#### 2.7.5. Extraction recovery and the matrix effect.

To evaluate extraction recovery, blank tissue samples were prepared in two manners: one was spiked (to obtain the concentration of all standards at 1 ng/mL) with the four standards before sample processing (sample A), and the other stood clean (sample B). Both sample types were prepared in triplicate. Then, all samples were incubated on ice (for 30 min) before the subsequent steps of the procedure described in section 2.5 were performed. The four standards were added to sample B (clean) after its filtration. The recovery was calculated as a comparison of the mean peak area of response obtained for sample A to that obtained for sample B.

The matrix effect was also calculated. For this purpose, an additional sample was prepared (sample C). All four tested standards (at the same concentration as for sample B) were added to the MeOH/EtOH mixture and the ammonium acetate buffer (0.5M) (1:1). Then, the responses of each standard (area) in sample B were compared with the signals obtained for sample C.

## 3. Results and discussion

### 3.1. Performance of MS/MS

The method development began with the selection of precursor ions. All standards were analyzed in both ion modes. For SIM and LOV, the most intensive signals were formed through protonation in positive ion mode. While the most intensive signals for SIMA and LOVA were detected in the negative ion mode by deprotonation ([M-H]^-^). Then, the selected precursor ions were fragmented, and two (for SIMA and LOVA) or three (for SIM and LOV) product ions were chosen (Table 2). For each selective *m/z* precursor/product ions transition the collision energy was optimised. After setting the LC conditions, all ion source parameters were also optimized.

### 3.2. Performance of LC

The analytes were separated on the C18 column (equipped with a guard column), which was selected based on the literature describing the methods for SIM and SIMA determination in plasma samples [11,13,14,23]. The use of this column type yielded good-shape peaks. Both elution types, isocratic and gradients with different mobile phases, were tested to select the best conditions for analytes separation and signal intensities with acceptable total run time and peak shape (S2 Appendix, Table S1). All gradient compositions show good separation, while the isocratic elution – does not (S2 Appendix, Fig S1), which is interesting because almost all previously described methods for SIM and SIMA determination in plasma samples applied this type of elution [9,11,13,14,24]. To obtain a possibly short time of analysis and to minimize the influence of matrix components, a decision was made to use gradient elution. Additionally, using a broad range of organic phase percentages (5–95%) allows for avoiding problems with pressure in the system, which may increase when lipid-reach samples (tissue extracts) are analyzed. An ammonium acetate buffer was added to phase A for a few reasons. Namely, it was used to enhance the ionic strength of the eluent and to increase ionization efficiency. In addition, it is important to control the system pH. Both forms of determined compounds, lactone and acidic, tend to interconvert, depending on the actual pH and temperature conditions. Maintaining low temperature and pH in the range of 4–5 reduced this process [13]. For this purpose, a buffer was added to the mobile phase to control the conditions in the chromatographic system. Using a 50 mM ammonium acetate buffer in phase A resulted in the highest peak responses. Other tested buffer concentrations showed definitely lower analyte intensities (S2 Appendix, Fig S2). The analyzed compounds show good separation at the final chromatographic settings.

### 3.3. Method optimization

**Fig 2:**
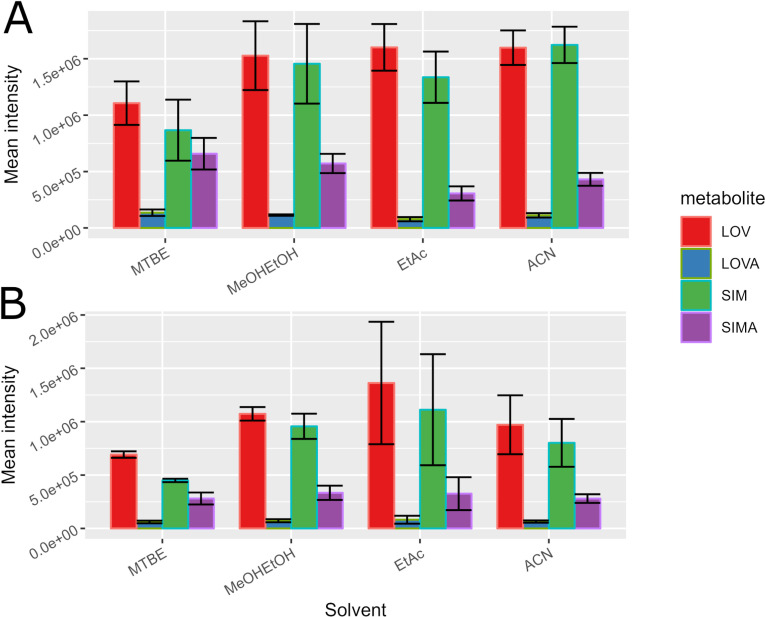
Solvents for homogenization and extraction selection. Panel A: without the ammonium acetate buffer. Panel B: ammonium acetate buffer-based homogenization connected with various agents used for extraction.

**Fig 3:**
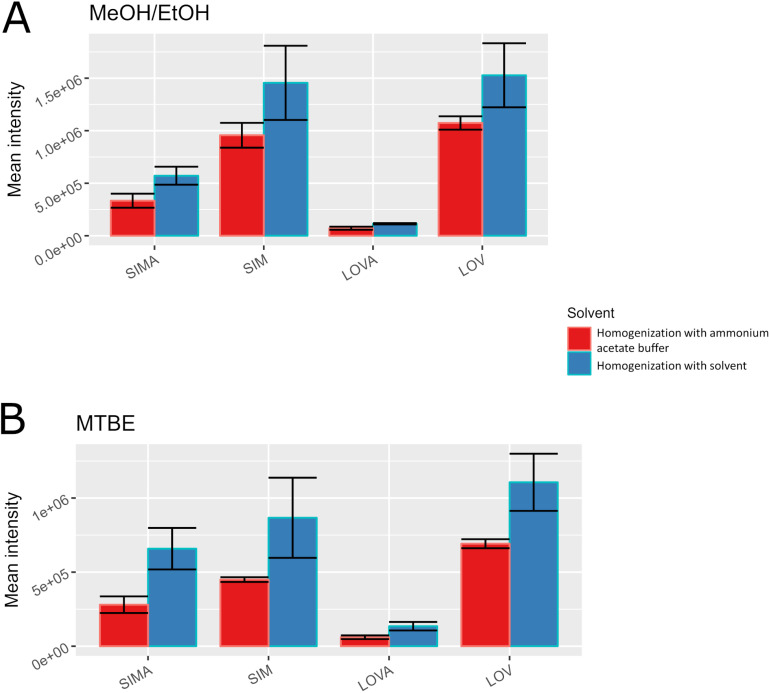
The influence of the controlled pH conditions at the sample preparation step on the obtained signals (panel A: extraction using a mixture of MeOH and EtOH; panel B: extraction using MTBE).

The first optimization step aimed to select the best solutions for homogenization and extraction. At the beginning, four solvents were tested: MTBE, MeOH/EtOH, EtAc, and ACN. The samples homogenized and extracted using ACN gave the highest mean intensity of response for all four compounds, with the highest repeatability among the compared solvents (Fig 2A). In general, the mean intensities of response obtained with EtAc and the MeOH/EtOH mixture were similar to the results obtained when ACN was used for homogenization and extraction, but their repeatability rates were lower. The use for homogenization of 0.5 M ammonium acetate buffer has an influence on the intensities and repeatability of obtained signals (Fig 2B). The same four solvents were tested for extraction purposes. Signal intensities slightly decreased for all tested solvents, while the results obtained with the MeOH/EtOH mixture and MTBE showed a significant repeatability increase (Fig 3). Among all buffer-homogenized samples, the one extracted with MTBE provided the lowest values of standard deviation and the lowest intensities. The most intense signals were obtained for EtAc extraction but with unacceptably low repeatability. For this reason, the extraction with the MeOH/EtOH mixture was chosen for the subsequent tests. This solvent yielded signal intensities similar to ACN extraction but with definitely better repeatability. Another advantage of MeOH/EtOH extraction is the exclusion of sample drying and reconstitution, which may be a source of additional analyte losses during sample preparation.

**Fig 4:**
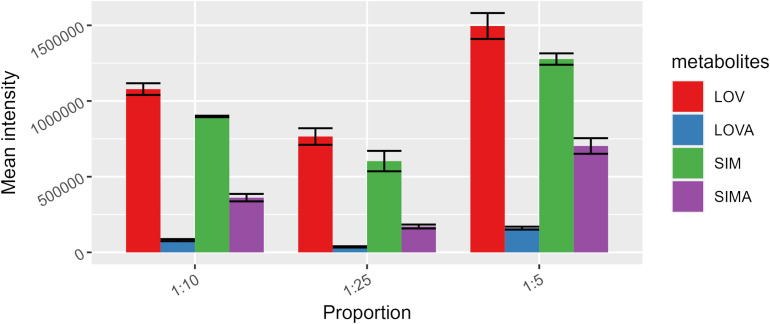
Mean signal intensities obtained for each analyte at three different tissue-to-solvent volume proportions.

Developing the sample preparation procedure also requires optimizing the solvent volume used for homogenization and extraction. Three proportions were tested: 5, 10, and 25 µL per 1 mg of tissue. Table 3 presents the RSD results obtained for each proportion. All these values are acceptable (≤11.2%). Therefore, the selection of the proper proportion was based on the mean signal intensity (Fig 4). The use of 5 µL per 1 mg of tissue showed a definitely higher signal than the other volumes used.

**Table 3:** The RSD results were obtained for different proportions of the extraction solvent/tissue (each proportion was prepared in triplicate repetition, n = 3).

Proportion	SIM	SIMA	LOV	LOVA
5 µL per 1 mg	2.9%	7.3%	5.7%	6.0%
10 µL per 1 mg	0.5%	6.8%	3.6%	8.6%
25 µL per 1 mg	11.2%	7.5%	7.1%	11.2%

### 3.4. Method validation

#### 3.4.1. Selectivity and sensitivity.

**Fig 5:**
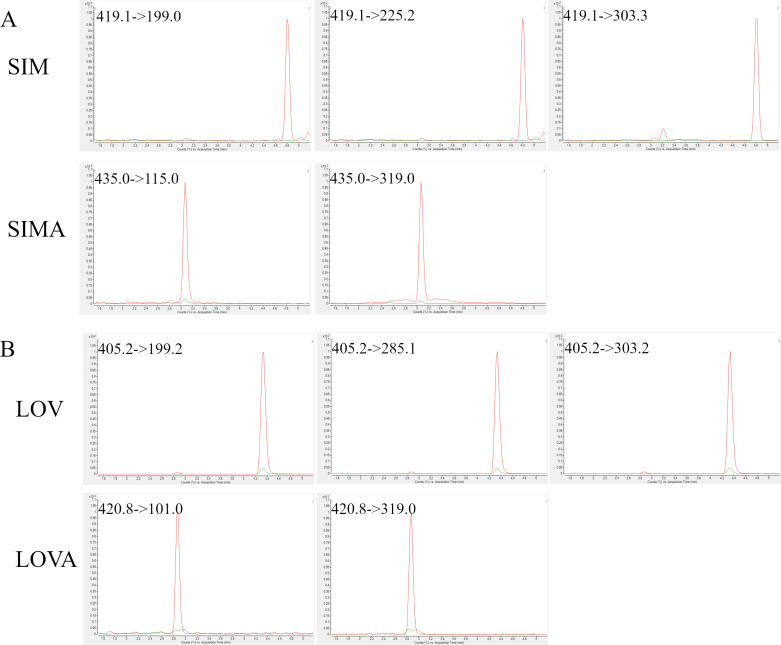
Chromatograms proved the selectivity of the proposed method. Red line: tissue spiked at the LOQ level; green line: blank tissue. Panel A: SIM and SIMA; panel B: LOV and LOVA.

**Fig 6:**
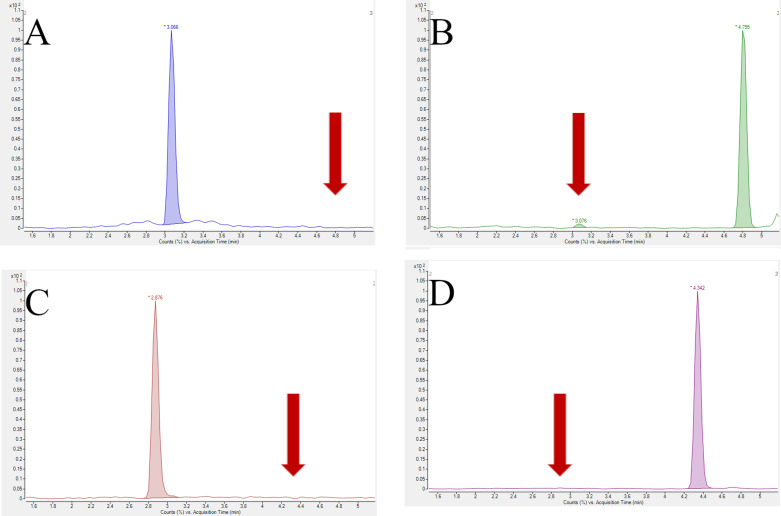
Chromatograms presented the specificity of the proposed method. They show a lack of the signal that came from SIMA in the RT of SIM (panel A) and vice versa (panel B), and the same for the signal that came from LOVA in the RT of LOV (panel C) and vice versa (panel D).

The chromatogram inspection showed good analytes separation of all tested compounds. A comparison of the signals that came from the tissue sample spiked with the standards at the LOQ level with the blank tissue sample proved good method selectivity: no endogenous substances were found to interfere with the analytes (Fig 5). Moreover, the in-source conversion SIM/SIMA, described by Ahmed et. al [13], was not observed in our study (Fig 6).

The LOQ was tested at additional lower levels (the entire linearity range tested: 0.01–20.0 ng/mL). For SIM and SIMA, a concentration of 0.1 ng/mL met the FDA acceptance criteria for the LOQ. The accuracy values were 98% and 107% for SIM and SIMA, respectively. This level of method sensitivity is comparable to other similar LC-MS methods for SIM and SIMA determination in plasma samples [11,14].

#### 3.4.2. Linearity, precision and accuracy.

Six-point calibration curves were obtained by plotting the ratio of the analyte to the IS peak area versus the nominal concentration. The weighted (1/X) linear regression was used for both analytes to calculate the calibration curve parameters such as slope, intercept, and correlation coefficient. The concentration range (0.1–5 ng/mL) displayed good linearity (the correlation coefficient for SIM was R^2^ = 0.9927 ± 0.0041 and for SIMA R^2^ = 0.9961 ± 0.0032) which was described by equations: y = 0.1864(±0.0864)x + 0.0112(±0.0045) for SIM, and y = 0.2704(±0.0667)x + 0.0428(±0.0074) for SIMA. A narrower concentration range was selected than in other similar methods [11,13,14] because muscle tissue was expected to have only a trace amount of statins (the majority is transferred via the bloodstream into hepatic cells – the first pass effect). The observed mean back-calculated concentrations, including precision and percent relative errors, are presented in Table 4.

**Table 4:** A summary of calibration standards (n = 3).

Analyte	Nominal concentration [ng/mL]	Mean^a^ ± SD [ng/mL]	Relative error [%]
SIM	0.1	0.098 ± 0.016	–1.7
	0.2	0.214 ± 0.013	7.1
	0.5	0.544 ± 0.063	8.8
	1.0	1.060 ± 0.130	6.0
	2.0	1.722 ± 0.273	–13.9
	5.0	5.002 ± 0.083	0.0
SIMA	0.1	0.107 ± 0.021	7.4
	0.2	0.174 ± 0.006	–12.8
	0.5	0.521 ± 0.089	4.2
	1.0	1.029 ± 0.052	2.9
	2.0	1.868 ± 0.046	–6.6
	5.0	5.099 ± 0.065	2.0

^a^Mean of three replicates at each concentration.

The precision and accuracy of the described method were determined by inter- and intra-day assays. The RSD of the obtained results ranged from 0.8 to 12.9%, whereas the mean accuracy values were between 91.4 and 115.4% (Table 5). These results prove that the method is precise and allows obtaining accurate results.

**Table 5:** Intra- and inter-day precision and accuracy.

Analyte	Nominal concentration [ng/mL]	Intra-day (n = 4)		Inter-day (n = 4)	
		Accuracy [mean ± SD, %]	Precision [% RSD]	Accuracy [mean ± SD, %]	Precision [% RSD]
SIM	5.0	91.4 ± 10.3	6.0	100.4 ± 7.3	6.9
	1.0	105.0 ± 8.5	7.5	98.9 ± 8.7	14 0
	0.2	96.0 ± 5.8	0.8	97.6 ± 9.7	14.0
SIMA	5.0	102.2 ± 6.9	12.9	115.4 ± 9.4	8.1
	1.0	92.6 ± 8.0	7.3	94.1 ± 7.1	10.9
	0.2	95.8 ± 6.5	3.6	94.2 ± 11.0	11.7

#### 3.4.3. Carry-over.

The comparison of signals obtained from blank samples and LOQ level does not show a significant carry-over for SIM and SIMA (6.2% and 17.3% respectively). However, results obtained for LOV (6.2%) and LOVA (9.0%) were not accepted for compounds used as IS. Therefore it was decided to inject blank samples after each analytical sample.

#### 3.4.4. Extraction recovery and the matrix effect.

The efficiency of the extraction procedure was evaluated by calculating the recovery of a particular sample preparation procedure, i.e., homogenization with the 0.5M ammonium acetate buffer and extraction with the MeOH/EtOH mixture. The following values were obtained: 76% ± 14% for SIM, and 99% ± 17% for SIMA. Combining these values with sufficient repeatability and signal intensity (Fig 4) gives reliable results.

Another crucial parameter, especially in the case of extraction from muscle tissue, is the matrix effect. The metabolite-rich (mainly for lipids) matrix type may have a potentially high influence on signal intensity (both *via* suppression or enhancement). Most methods for SIM and SIMA determination in plasma used extraction with MTBE [24,30] or diethyl ether [13], without any significant matrix effect. In the case of the presented study, ethyl acetate gave unacceptable repeatability (even in homogenization with the ammonium acetate buffer). In contrast, MTBE showed much better repeatability but with lower total signal intensities (Fig 3). Considering the above, the MeOH/EtOH mixture was tested for extraction, and the matrix effect for this mixture was determined with the following results: 87% ± 5% for SIM, and 139% ± 4% for SIMA. The influence of the matrix components on the SIMA results is quite high, therefore it was decided to determine the normalized matrix effect [31,32]. For this purpose, the matrix effect was additionally determined for LOVA and LOV. As a result were obtained values: 120% ± 15% (LOVA) and 77% ± 3% (LOV). The normalized matrix effect was calculated as a ratio of the matrix effect obtained for the analyte to ISs’ matrix effect. An accepted result should be between 0.8 and 1.2. In the presented data, the normalized matrix effect for SIMA was 1.16 ± 0.09, and for SIM was 1.12 ± 0.08. Both values were in the acceptance range.

## 4. Real sample analysis

**Fig 7:**
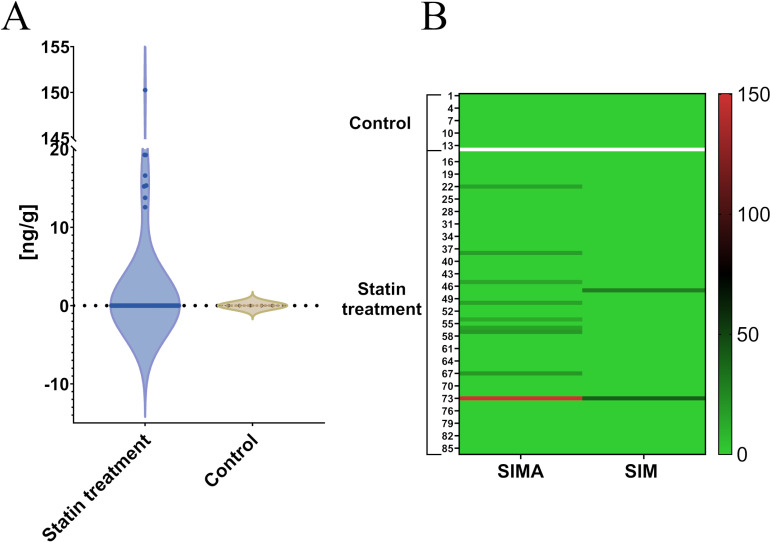
SIM and SIMA detection in real samples. Panel A: violin plot of SIMA distribution in control and statin-treated patient groups; panel B: the heatmap of SIM and SIMA detection in both study groups.

The analysis of samples obtained from statin-treated patients proves that the proposed method allows the determination of SIM and SIMA in skeletal muscle tissue. In nine of 86 tested samples, SIMA was determined, and in two samples – SIM. As expected, all samples obtained from the control group (without statin-based treatment) gave results below LOQ (Fig 7A). At the same time in the samples obtained from 9 patients in muscle tissue SIMA was in the range 12.58–19.26 ng/g. One of all tested samples contains a significantly higher SIMA concentration (150.26 ng/g). This result was unexpected, however similar abnormality was detected also in the plasma samples obtained from this patient. At least one of the determined two statin forms was detected in 10 of 73 (14%) samples obtained from patients treated with statin (Fig 7B). It is known that SIM may accumulate in the different solid tissues [33–35] including skeletal muscle. However, as a result of the high first-pass effect the highest concentration is expected (and observed) in the liver [25,34]. A small amount of all absorbed drugs’ dose is available to accumulate in other tissues. This probably may be a reason, why SIM and SIMA were detected in only around 14% of tested samples. It is proved, that statin accumulation in other than liver tissues may increase when additional medication is applied [22]. These are mainly drugs metabolized by the cytochrome P450 enzymes family, which are also crucial for SIM metabolizing. However, in the presented study patients treated with other CYP450-metabolized medications were excluded from the study. So, the explanation of the reasons and consequences of SIM and SIMA detection in skeletal muscle needs a more detailed study at the metabolome level.

## 5. Conclusions

In conclusion, a reliable LC-MS method for the SIM and SIMA determination in muscle tissue samples was developed. Such parameters as precision, selectivity, sensitivity, and accuracy met the criteria defined in ICH M10 guidelines 2023 and FDA 2018 guidelines. Additionally, the matrix effect, recovery, and carry-over of the proposed method were acceptable. The using of the method for the determination of SIM and SIMA in real samples proved its applicability for samples obtained from statin-treated patients. This is the first study presenting the determination of the selected statin and its metabolite in muscle tissue. The presented method may be a tool for explaining many intriguing aspects of statin therapy.

## Supporting information

S1 Appendix Description of the analytical method used for the determination of SIM and SIMA in plasma samples. (DOCX)

S2 Appendix Details of the tested elution types and mobile phase composition. (DOCX)
