# Supplementary material for: Development of an LC-MS method for the determination of simvastatin and its hydroxy acid form in muscle tissue and method application
Source: PLoS One. 2025 May 5;20(5):e0322808. doi: 10.1371/journal.pone.0322808 (PMC12052154; doi:10.1371/journal.pone.0322808)
Supplement: S1 Appendix — (DOCX) [file pone.0322808.s001.docx]

**Methods for determination of SIM and SIMA in plasma samples using LC-MS/MS system**

The concentration of SIM and SIMA in plasma samples was determined using an in-house method developed based on the available literature [1-4].

1. Chemicals and reagents

Purified water was obtained using the Milli-Q Integral 3 system (Millipore SAS, Molsheim, France). Analytical standards of simvastatin (SIM, 99%) and lovastatin (LOV, EP reference standard), acetic acid (EP reagent grade), and ammonium acetate (LiChropur, an eluent additive for LC-MS) were purchased from Sigma-Aldrich Chemie GmbH (Steinheim, Germany). Simvastatin hydroxy acid (SIMA, analytical standard 99%), and lovastatin hydroxy acid sodium salt (LOVA, analytical standard 97%) were purchased from Biosynth Ltd (the United Kingdom). Ethyl acetate (EtAc) and acetonitrile (ACN) the Optima LC-MS grade were purchased from Thermo Fisher Scientific (San Jose, California, the USA).

1. LC-MS instrumentation

The LC-MS system was composed of a 1290 Infinity II UHPLC system, combined with a 6495 triple quadrupole mass spectrometer (both from Agilent Technologies, Santa Clara, California, the USA) equipped with the electrospray ionization (ESI AJS) ion source and iFunnel technology.

1. LC-MS conditions

Chromatographic separation was performed on an analytical column Zorbax RRHD Eclipse Plus C18 (2.1 × 50 mm, 1.8 µm), connected to a guard column Zorbax RRHD Eclipse Plus C18 (2.1 × 5 mm, 1.8 µm), thermostated at 30°C. The gradient elution with an ammonium acetate buffer (50 mM in water, pH 5.0 adjusted with acetic acid) as phase A, and pure ACN as phase B, was used to separate statins and the rest of the sample components. The separation was started at 5% B, then in 2 min was increased to 60% B, and further to 85% B in the next 3 min, to reach the final composition of 95% B at 5.2 min, and was then retained for 1.8 min (total run time: 7 min). After each run, the system was conditioned for 2 min with the initial mobile phase composition. The flow rate was 0.25 mL/min. The autosampler was maintained at 8°C, and the injection volume was 5 µL.

The mass spectrometer was operated in the multiple reaction monitoring mode (MRM) with switching polarization. SIM and LOV were observed in the positive ion mode, while SIMA and LOVA were in the negative ion mode. Following *m/z* transitions precursor/product ion were observed (with collision energies in the brackets): 419.1/199.2 (30 V), 419.1/225.2 (32 V) and 419.1/303.3 (12 V) for SIM, 435.0/319.0 (20 V) and 435.0/115.0 (14 V) for SIMA, 405.2/285.1 (10 V), 405.2/303.2 (10 V) and 405.2/199.2 (20 V) for LOV, and 420.8/319.0 (18 V) and 420.8/101.0 (40 V) for LOVA.

The ion source conditions were as follows: the gas temperature was set at 290°C with a 15 L/min gas flow rate, and the sheath gas temperature and flow rate at 350°C and 11 L/min, respectively. The nebulizer worked at 40 psi, the nozzle voltage was 0 V, and the capillary voltages were 3000 V for the positive and 5500 V for the negative ion modes.

1. Calibration

The method was linear in the 0.5 – 25 ng/mL (for both SIM and SIMA). The calibration curve was constructed based on measuring the samples at 7 calibration levels. Samples for calibration and quality control (QC) were prepared by spiking the working solution of all four standards (SIM, SIMA, LOV, and LOVA) into statin-free plasma samples.

1. Samples collection

Plasma samples were obtained from hypercholesterolemic patients either at the diagnosis moment, before starting the statin treatment (control group), or already statin-treated patients (study group). The daily dose of simvastatin was 40 mg. Blood samples were collected in heparin-coated vacutainers. Blood cells were separated by centrifugation, and then portions of plasma samples were collected and stored at -80°C until the analysis day.

1. Samples preparation

On the day of analysis, samples were thawed on ice. Then to the portion of 200 µL of plasma samples, 13.1 µL of the LOV and LOVA solution (IS solution, concentration in the mixture of water and ACN (7/3 v/v) of both compounds was 500 ng/mL), 100 µL of 0.5 M ammonium acetate buffer (to control sample pH), and 1 mL of ethyl acetate (extraction agent). After vortex mixing (by 3 min) this mixture was centrifuged by 20 min at 21 000 × g at 4°C. Then, 900 µL of the organic layer was transferred into a vial and evaporated to dryness at room temperature using a vacuum concentrator (SpeedVac Thermo Fisher Scientific, San Jose, California, the USA). In the end, the sample was reconstituted in 100 µL of a mixture of water and ACN (7/3 v/v) and analyzed with an LC-MS system.

1. Literature

[1] T.A. Ahmed, J. Horn, J. Hayslip, M. Leggas, Validated LC-MS/MS method for simultaneous determination of SIM and its acid form in human plasma and cell lysate: Pharmacokinetic application, J Pharm Anal 2(6) (2012) 403-411. https://doi.org/10.1016/j.jpha.2012.07.010.

[2] S.C.R. Silva, G.R.d. Rezende, V.B. Boralli, Quick and simple LC-MS/MS method for the determination of simvastatin in human plasma: application to pharmacokinetics and bioequivalence studies, Brazilian Journal of Pharmaceutical Sciences 50 (2014).

[3] S.B. Munaga, R.K. Valluru, P.B. Bonga, V.S. Rao, H.K. Sharma, Development and Validation of an LC-MS-MS Method for the Simultaneous Determination of Simvastatin, Simvastatin Acid and Ezetimibe in Human Plasma and Its Application to Pharmacokinetic Study in the Indian Population, J Chromatogr Sci 54(6) (2016) 985-96. https://doi.org/10.1093/chromsci/bmw043.

[4] A. El-Zailik, L.K. Cheung, Y. Wang, V. Sherman, D.S. Chow, Simultaneous LC-MS/MS analysis of simvastatin, atorvastatin, rosuvastatin and their active metabolites for plasma samples of obese patients underwent gastric bypass surgery, J Pharm Biomed Anal 164 (2019) 258-267. https://doi.org/10.1016/j.jpba.2018.10.045.
