# Supplementary material for: Development of an LC-MS method for the determination of simvastatin and its hydroxy acid form in muscle tissue and method application
Source: PLoS One. 2025 May 5;20(5):e0322808. doi: 10.1371/journal.pone.0322808 (PMC12052154; doi:10.1371/journal.pone.0322808)
Supplement: S2 Appendix — (DOCX) [file pone.0322808.s002.docx]

**Additional results of the LC-MS method optimization**

Chromatograms present the peak separation obtained with elution types described in the S1 table. For testing different elution conditions mixture of all four standards (at the concentration of 1 µg/mL) was injected (1µL) into the system.

**Table S1**. Details of the tested elution types and mobile phase compositions.

| Elution type | Phase A | Phase B | Mobile phase composition | | Flow rate [mL/min] | Figure No. |
| --- | --- | --- | --- | --- | --- | --- |
|  |  |  | min | %B |  |  |
| Isocratic | H_2_O+0.1%FA | ACN+0.1%FA | 0.0 | 70 | 0.5 | A |
|  |  |  | 15.0 | 70 |  |  |
| Gradient 1 | H_2_O+0.1%FA | ACN+0.1%FA | 0.0 | 0 | 0.5 | B |
|  |  |  | 14.0 | 100 |  |  |
| Gradient 2 | H_2_O+0.1%FA | ACN+0.1%FA | 0.0 | 5 | 0.5 | C |
|  |  |  | 6.0 | 50 |  |  |
|  |  |  | 8.0 | 60 |  |  |
|  |  |  | 10.0 | 95 |  |  |
| Gradient 3 | H_2_O+0.1%FA | ACN+0.1%FA | 0.0 | 5 | 0.5 | D |
|  |  |  | 2.0 | 50 |  |  |
|  |  |  | 5.0 | 60 |  |  |
|  |  |  | 7.0 | 95 |  |  |
| Gradient 4 | 50 mM ammonium acetate buffer | ACN | 0.0 | 5 | 0.25 | E |
|  |  |  | 2.0 | 60 |  |  |
|  |  |  | 5.0 | 85 |  |  |
|  |  |  | 5.2 | 95 |  |  |
|  |  |  | 9.0 | 95 |  |  |


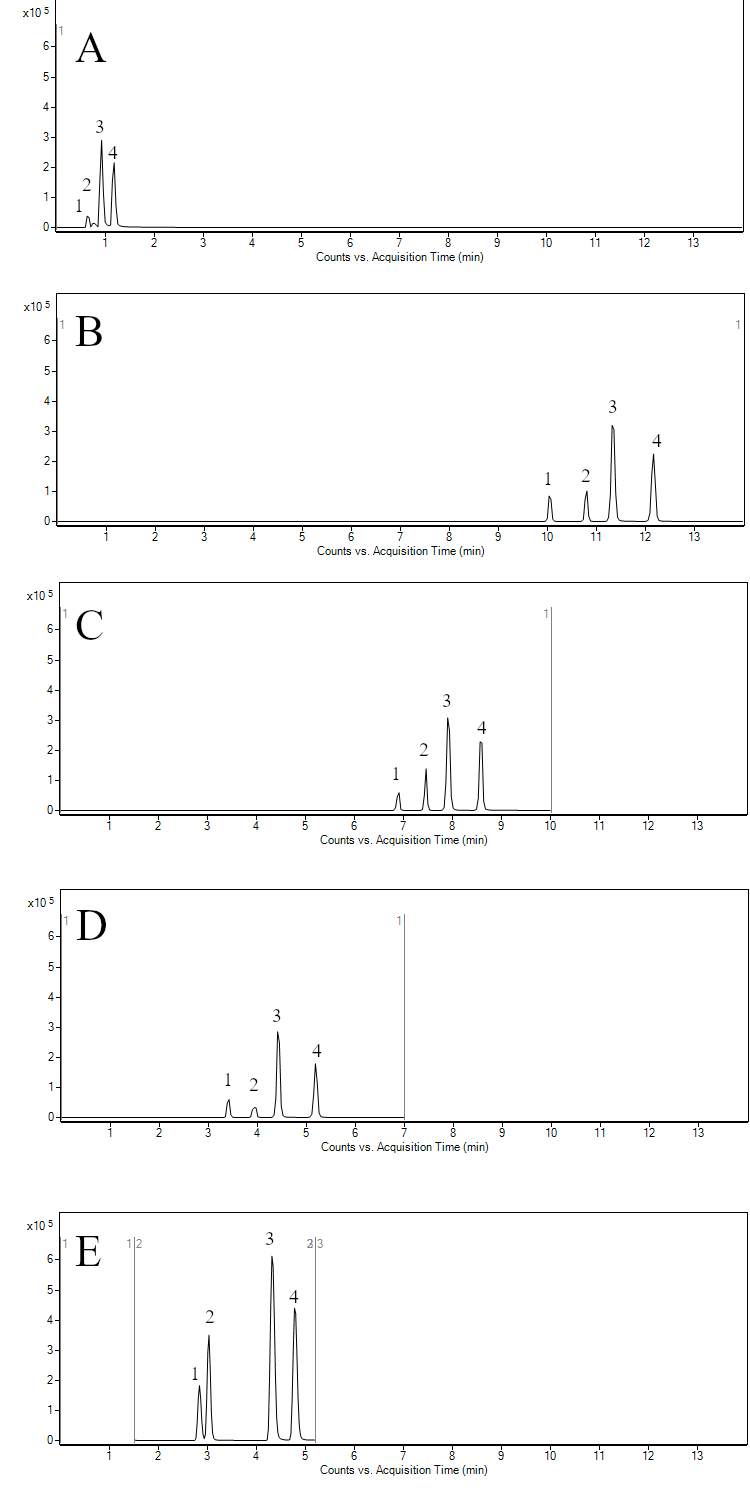
**Fig S1**. Chromatographic separation in different elution types and mobile phase composition. 1: LOVA, 2: SIMA, 3: LOV, 4: SIM.

The comparison of the buffers working as Phase A with different concentrations. For each analysis, 1 µL of the standard mixture (1 µg/mL) was injected into the system.


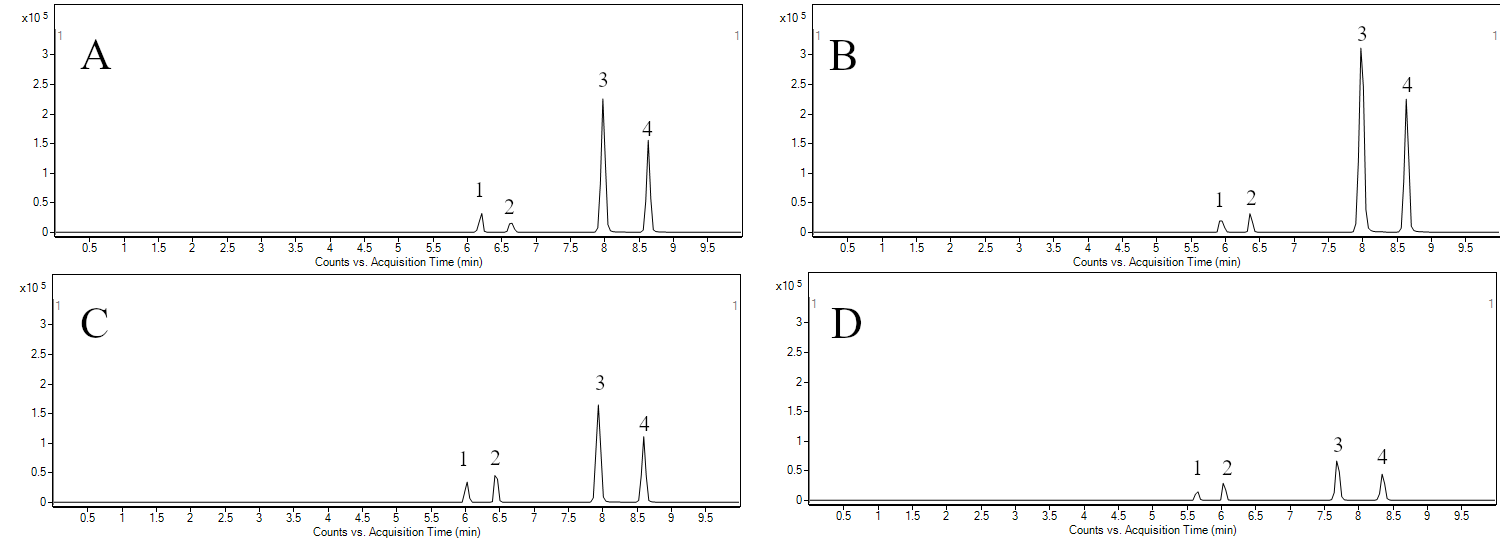


**Fig S2**. Chromatograms were obtained with the analysis of the mixture of standards (1: LOVA, 2: SIMA, 3: LOV, 4: SIM) with ammonium acetate buffer with different concentrations: A: 2 mM ammonium acetate buffer, B: 10 mM ammonium acetate buffer, C: 100 mM ammonium acetate buffer, D: 500 mM ammonium acetate buffer. Chromatograms were collected with sample analyzing using Gradient 2.
